# Optical assessment of scars after endoscopic mucosal resection of large colorectal polyps in a multicenter, community hospital setting: is routine biopsy still necessary?

**DOI:** 10.1055/a-2498-7114

**Published:** 2025-01-28

**Authors:** Lonne W. T. Meulen, Roel M. M. Bogie, Peter D. Siersema, Bjorn Winkens, Marije S. Vlug, Frank H. J. Wolfhagen, Martine A. M. C. Baven-Pronk, Michael P. J. A. van der Voorn, Matthijs P. Schwartz, Lauran Vogelaar, Tom C. J. Seerden, Wouter L. Hazen, Ruud W. M. Schrauwen, Lorenza Alvarez Herrero, Ramon-Michel Schreuder, Annick B. van Nunen, Gijs J. de Bruin, Willem A. Marsman, Marc de Bièvre, Robert Roomer, Rogier J.J. de Ridder, Maria Pellisé, Michael J. Bourke, Ad A. M. Masclee, Leon M. G. Moons, on behalf of the OPTICAL-STAR Working Group, Yasser A. Alderlieste, Alaa Alkhalaf, Marloes Bigirwamungu-Bargeman, Femke Boersma, Philip Bos, John Groen, Edith Kuiper, Monigue E. van Leerdam, Jos M. Ramaker, Linda B. J. Roberts-Bos, Esther Stoop, Karsten Thurnau, Roland de Vries

**Affiliations:** 1 Department of Gastroenterology and Hepatology, Maastricht University Medical Center, Maastricht, The Netherlands; 2 GROW, School for Oncology and Reproduction, Maastricht University, Maastricht, The Netherlands; 3 Department of Gastroenterology and Hepatology, Erasmus University Medical Center, Rotterdam, The Netherlands; 4 Department of Methodology and Statistics, Maastricht University, Maastricht, The Netherlands; 5 CAPHRI, Care and Public Health Research Institute, Maastricht University, Maastricht, The Netherlands; 6 Department of Gastroenterology and Hepatology, Dijklander Hospital, Hoorn, The Netherlands; 7 Department of Gastroenterology and Hepatology, Albert Schweitzer Hospital, Dordrecht, The Netherlands; 8 Department of Gastroenterology and Hepatology, Groene Hart Hospital, Gouda, The Netherlands; 9 Department of Gastroenterology and Hepatology, Haga Hospital, Den Haag, The Netherlands; 10 Department of Gastroenterology and Hepatology, Meander Medical Center, Amersfoort, The Netherlands; 11 Department of Gastroenterology and Hepatology, Diakonessenhuis, Utrecht, The Netherlands; 12 Department of Gastroenterology and Hepatology, Amphia Hospital, Breda, The Netherlands; 13 Department of Gastroenterology and Hepatology, Elisabeth-Tweesteden Hospital, Tilburg, The Netherlands; 14 Department of Gastroenterology and Hepatology, Bernhoven, Uden, The Netherlands; 15 Department of Gastroenterology and Hepatology, Sint Antonius Hospital, Nieuwegein, The Netherlands; 16 Department of Gastroenterology and Hepatology, Catharina Hospital Eindhoven, Eindhoven, The Netherlands; 17 Department of Gastroenterology and Hepatology, Zuyderland Medical Center, Sittard-Geleen, The Netherlands; 18 Department of Gastroenterology and Hepatology, Tergooi Hospital, Hilversum, The Netherlands; 19 Department of Gastroenterology and Hepatology, Spaarne Gasthuis, Haarlem, The Netherlands; 20 Department of Gastroenterology and Hepatology, Viecuri Medical Center, Venlo, The Netherlands; 21 Department of Gastroenterology and Hepatology, Franciscus Gasthuis and Vlietland, Rotterdam, The Netherlands; 22 Department of Gastroenterology, Hospital Clínic de Barcelona, Barcelona, Spain; 23 Department of Gastroenterology and Hepatology, Westmead Hospital and Westmead Clinical School, University of Sydney, Sydney, New South Wales, Australia; 24 Department of Gastroenterology and Hepatology, University Medical Center Utrecht, Utrecht, The Netherlands; 25 Department of Gastroenterology and Hepatology, Rivas, Gorinchem, The Netherlands; 26 Department of Gastroenterology and Hepatology, Isala Clinics, Zwolle, The Netherlands; 27 Department of Gastroenterology and Hepatology, Medical Spectrum Twente, Enschede, The Netherlands; 28 Department of Gastroenterology and Hepatology, Gelre Hospitals, Apeldoorn, The Netherlands; 29 Department of Gastroenterology and Hepatology, Gelderse Vallei Hospital, Ede, The Netherlands; 30 Department of Gastroenterology and Hepatology, Sint Jansdal Hospital, Harderwijk, The Netherlands; 31 Department of Gastroenterology and Hepatology, Maasstad Hospital, Rotterdam, The Netherlands; 32 Department of Gastroenterologic Oncology, The Netherlands Cancer Institute, Antoni van Leeuwenhoek Hospital, Amsterdam, and Department of Gastroenterology and Hepatology, Leiden University Medical Center, Leiden, The Netherlands; 33 Department of Gastroenterology and Hepatology, Elkerliek Hospital, Helmond, The Netherlands; 34 Department of Gastroenterology and Hepatology, Laurentius Hospital Roermond, Roermond, The Netherlands; 35 Department of Gastroenterology and Hepatology, Haaglanden Medical Center, Den Haag, The Netherlands; 36 Department of Gastroenterology and Hepatology, Hospital Group Twente, Almelo, The Netherlands; 37 Department of Gastroenterology and Hepatology, Deventer Hospital, Deventer, The Netherlands

## Abstract

**Background** Piecemeal endoscopic mucosal resection (EMR) of large (≥ 20 mm) nonpedunculated colorectal polyps (LNPCPs) is succeeded by a 6-month surveillance endoscopy to evaluate the post-EMR scar for recurrence. Data from expert centers suggest that routine tattoo placement and scar biopsies can be omitted, but data from community hospitals are lacking.

**Methods** The agreement between optical assessment and histological confirmation by routine biopsies was evaluated in a post-hoc analysis of the STAR-LNPCP study (NTR7477), containing prospective data on 6-month post-EMR scar assessments in 30 Dutch community hospitals (October 2019 to May 2022). A standardized protocol was followed for documentation of optical characteristics, imaging, and biopsy of the post-EMR scar.

**Results** In 1277 post-EMR scar assessments, identification of the scar was achieved in 1215/1277 (95 %). Tattoo placement did not influence scar identification. Scar biopsy was performed in 1050/1215 cases (86 %). Recurrences were seen in 200/1050 cases (19 %). There was good agreement between optical assessment of recurrence and histological confirmation (Cohen’s kappa 0.78 [95 %CI 0.73–0.83]). The negative and positive predictive values were 98 % (95 %CI 97 %–99 %) and 74 % (95 %CI 68 %–80 %), respectively. A higher false-positive rate was seen after prior use of clips (11 % vs. 5 %; *P* = 0.02). Dedicated endoscopists identified the scar more often (96 % vs. 88 %; *P* < 0.001), and showed a lower optical recurrence miss rate (1 % vs. 3 %; *P* = 0.11) compared with nondedicated endoscopists.

**Conclusion** Based on this multicenter community hospital study, routine tattoo placement and scar biopsies of the post-EMR scar can be omitted. Assessment of post-EMR scars by dedicated endoscopists is advised.

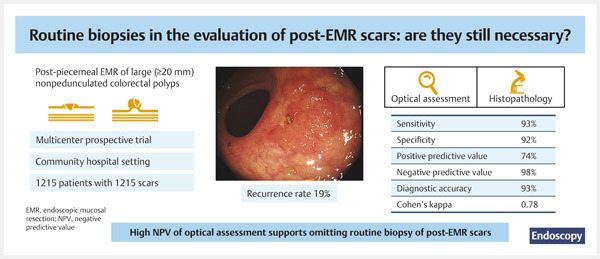

## Introduction

The most commonly used treatment modality for noninvasive large ( ≥ 20 mm) nonpedunculated colorectal polyps (LNPCPs) is endoscopic mucosal resection (EMR). A first surveillance colonoscopy after 6 months to check for local recurrence is advocated in several guidelines 1 2 3 . Until recently, guidelines recommended routine biopsies of the post-EMR scar to confirm the absence of recurrence, and placement of a tattoo for post-EMR scar identification 2 4 . The recently updated European Society of Gastrointestinal Endoscopy (ESGE) guideline stated that routine biopsies can be omitted if sufficiently trained endoscopists have evaluated the scar tissue with enhanced imaging 1 , using a standardized imaging protocol 5 6 . Importantly, detection of the post-EMR scar was possible with easy-to-use optical evaluation criteria, without the need for universal tattoo placement 7 .

However, it remains uncertain whether these results can be extrapolated to nonexpert centers 8 . Therefore, in the setting of a prospective multicenter study 9 , we investigated whether the diagnostic accuracy of optical assessment of post-EMR scars for recurrence was high enough at a community level to refrain from standardized biopsies and the need for universal tattoo placement.

## Methods

In this post-hoc analysis of the STAR-LNPCP study (NTR7477), follow-up colonoscopies performed after EMR of an LNPCP in 30 Dutch community hospitals between October 2019 and May 2022 were included. The STAR-LNPCP study was a multicenter, cluster randomized trial, in which 59 endoscopists from 30 community hospitals included all consecutive LNPCPs. Participating hospitals were randomly chosen and were asked to nominate 1 or 2 candidates from their endoscopists who were dedicated to large-sized EMR. Randomization into the training or control group was performed on a cluster (center) level. Endoscopists from 15 centers were additionally trained in EMR of LNPCPs, while the endoscopists at the other 15 centers were not. Further study details are described in the original article by Meulen et al. 9 . The study was approved by the Medical Ethical Review Committee of the Maastricht University Medical Center (MEC 2017–0017). All patients provided written informed consent prior to the study.

### Patient selection

**Fig. 1:**
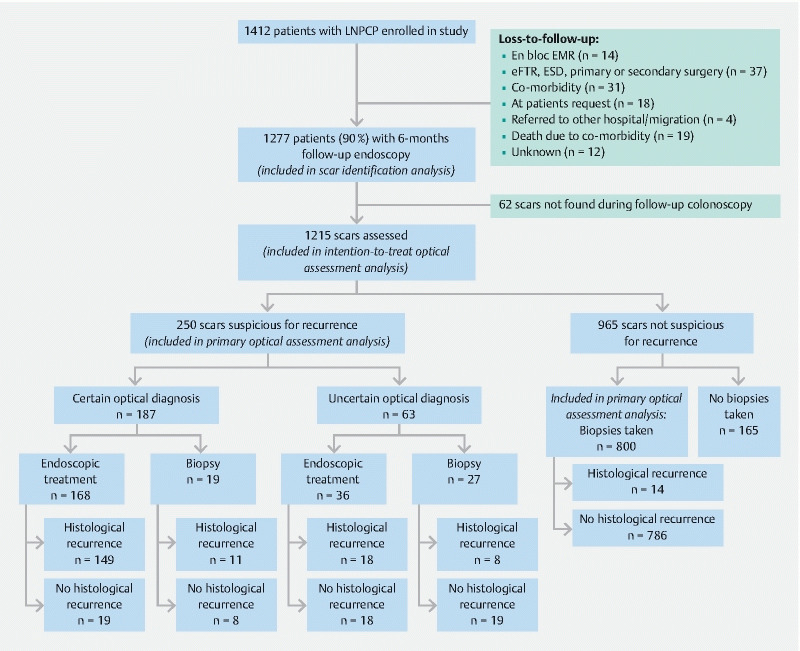
Flowchart of patient inclusion and outcomes. LNPCP, large nonpedunculated colorectal polyp; EMR, endoscopic mucosal resection; eFTR, endoscopic full-thickness resection; ESD, endoscopic submucosal dissection.

Consecutive patients undergoing follow-up colonoscopies after previous EMR of an LNPCP were included. Exclusion criteria were: initially incomplete EMR, inflammatory bowel disease, and poor bowel preparation (Boston Bowel Preparation Score < 2 for the segment of concern). All patients who underwent a 6-month surveillance colonoscopy in the STAR-LNPCP study were included in this post-hoc analysis ( Fig. 1 ).

### Baseline characteristics

Patient characteristics such as age, sex, American Society of Anesthesiologists (ASA) classification, medication use, and medical history were obtained from case record forms. Baseline lesion and treatment characteristics were obtained from endoscopy and histology reports. Baseline lesion characteristics consisted of size, morphology, location, accessibility, enhanced imaging, and initial histology. Baseline treatment characteristics consisted of EMR type (en bloc or piecemeal), use of adjunctive treatment (e. g. hot avulsion, cold avulsion, snare-tip soft coagulation, argon plasma coagulation), use of adjuvant thermal ablation, use of clips, and tattoo placement. When a patient had more than one LNPCP, only one LNPCP per patient was randomly included in the original study 9 .

### E-module assessment of post-EMR scar

Before starting the study, participating endoscopists had the opportunity to watch an e-module regarding the identification and assessment of post-EMR scars. In this e-module, the criteria for scar identification, standardized scar assessment, and biopsy protocol were explained. Furthermore, the difference between recurrence and post-EMR scar clip artifact was demonstrated by discussing several examples. The e-module topics and order, as well as some example images, are presented in **Appendix 1 s** , see online-only Supplementary Material.

### Standardized assessment of the EMR scar and biopsy protocol

A standardized protocol was followed during the assessment of the EMR scar. This included in vivo evaluation of the scar and potential recurrent neoplasia, with the combined use of white-light imaging, advanced imaging, and zoom/near focus, and pictures taken for every imaging modality. The scar was carefully assessed for recurrent neoplasia and the following characteristics of the scar were documented: location of the scar, size of the scar, presence of recurrence, certainty (yes/no) about the presence/absence of recurrence, unifocal or multifocal recurrence, location of recurrence (at the edge, in the center, or both), and the morphology of recurrence. When recurrence was present, this was treated and documented in the endoscopy report.

When there were no signs of recurrence, biopsies of the EMR scar were taken according to a standardized biopsy protocol: depending on the size and shape (e. g. straight line or round) of the scar, 1–3 biopsies were taken from the center of the scar, and in the periphery of the scar at least one biopsy per quadrant was performed. Biopsies from the center and periphery of the EMR scar were presented separately for histological evaluation in the pathology lab.

### Tattoo placement

Placement of a tattoo on the contralateral side of the post-EMR defect was left at the discretion of the endoscopist during the initial colonoscopy.

### Outcomes

The primary outcome was optical assessment of recurrence. Furthermore, the diagnostic accuracy, sensitivity, specificity, positive predictive value (PPV), and negative predictive value (NPV) were calculated. The predictive value of tattoo placement for identification of the scar was also evaluated.

In addition, differences in post-EMR scar identification between dedicated and nondedicated endoscopists were evaluated. Dedicated endoscopists were defined as endoscopists participating in the STAR-LNPCP study and performing large polypectomies in their center. Nondedicated endoscopists were defined as the endoscopists not participating in the STAR-LNPCP study and not primarily performing large polypectomies in their center. At the start of the STAR-LNPCP study, participating centers were asked which doctors performed large EMRs in their center.

Furthermore, the effect of prior clipping on the optical assessment of post-EMR recurrence was evaluated, with the hypothesis being that clipping would lead to post-EMR scar clip artifacts, which would be mistaken for recurrence. To evaluate this hypothesis, the PPV of clipped EMR defects was compared with that of nonclipped EMR defects.

The optical recurrence miss rate was defined as all histologically confirmed recurrences that were optically assessed as negative for recurrence, calculated as a proportion of the total number of scar inspections performed. False positives were defined as all optically assessed recurrences, that were not confirmed as a recurrence by histology, calculated as a proportion of total scar assessments.

### Statistical analysis

For descriptive statistics, categorical variables are presented as numbers and percentages, and numerical variables are presented as mean (SD) or median with interquartile range (IQR; 25th to 75th percentile). Pearson’s chi-squared or Fisher’s exact tests were used to compare groups regarding categorical variables.

Risk regression with correction for clustering of patients within endoscopists (generalized estimating equation [GEE] with exchangeable covariance structure and probit link) was performed to evaluate which variables (dedicated or nondedicated endoscopist, submucosal tattoo, size of initial LNPCP, location, accessibility, morphology) were independently related to the identification of the post-EMR scar. Furthermore, the same analysis was performed to evaluate the effect of clipping on optical recurrence assessment, with correction for dedicated vs. nondedicated endoscopists. Alongside the intraclass correlations obtained from these GEE analyses, we report the adjusted risk ratios (RR) with corresponding 95 %CI, and *P* values for each risk factor, corrected for all other risk factors in the model. As this included multiple testing, the Bonferroni corrected 95 %CIs and *P* values are also provided.

Cohen's kappa was used to determine the agreement between the optical assessment of recurrence and histological evaluation of biopsies. According to the definition proposed by Landis and Koch, a kappa of 0.61–0.80 was considered “moderate to good” and a kappa of 0.81–1.00 was considered “(almost) perfect” 10 . Furthermore, we calculated the NPV, PPV, sensitivity, specificity, and overall diagnostic accuracy with 95 %CIs. Analyses were performed in a per-protocol manner, including only scars that were found, assessed, and biopsied. Additionally, intention-to-treat analysis was performed, assuming that scars that were not found and scars that were not biopsied would not have shown any signs of recurrence when histological evaluation of biopsy had taken place.

Furthermore, sensitivity analysis with cluster bootstrapping was performed, where correction for clustering of patients/scars within the same endoscopist was made. A two-sided *P* value of ≤ 0.05 (after Bonferroni correction) was considered statistically significant.

Statistical analysis was performed using IBM SPSS Statistics v27.0.0, except for cluster bootstrapping, which was performed using R (v4.3.1). CIs for proportions (with continuity correction) were computed using http://vassarstats.net/prop1.html .

## Results

### Baseline characteristics

A total of 1277 patients (mean age 68 [SD 9]; 45 % women) who underwent 6-month surveillance colonoscopy after complete EMR of an LNPCP (median size 30 mm, IQR 25–40 mm; 64 % located in the proximal colon) were included ( Table 1 ). Surveillance colonoscopy and assessment of the post-EMR scar was performed by 161 endoscopists in 30 community hospitals, of whom 59 (37 %) were dedicated endoscopists.

**Table 1:** Characteristics of the 1277 included patients and their index colonoscopies.

	n (%) unless otherwise specified
**Patient characteristics**	
Age, mean (SD), years	68 (9)
Sex, female	573 (45 %)
ASA classification	
ASA I	238 (19 %)
ASA II	871 (68 %)
ASA III	167 (13 %)
ASA IV	1 (0 %)
**Index colonoscopy characteristics**	
**Indication for colonoscopy**	
Bowel cancer screening program	538 (42 %)
Referred	199 (16 %)
Surveillance	184 (14 %)
Symptomatic	356 (28 %)
BBPS ≥ 2 per inspected segment	1253 (98 %)
**Lesion characteristics**	
**Polyp location**	
Proximal	820 (64 %)
Distal	457 (36 %)
Polyp size, median (IQR), mm	30 (25–40)
**Size groups, mm**	
20–29	399 (31 %)
30–39	399 (31 %)
≥ 40	479 (38 %)
**Morphology**	
Sessile	789 (62 %)
Flat	488 (38 %)
**Accessibility**	
Easy	1111 (87 %)
Difficult	166 (13 %)
**SMSA score**	
II	64 (5 %)
III	586 (46 %)
IV	627 (49 %)
Clip placement	258 (20 %)
Submucosal tattoo	488 (38 %)

ASA, American Society of Anesthesiologists; BBPS, Boston Bowel Preparation Scale; IQR, interquartile range; SMSA: size, morphology, site, and access.

A total of 1215 scars were identified and assessed for the presence/absence of recurrence ( Fig. 1 ). In 1060 /1215 cases (87 %, 95 %CI 85 %–89 %), a dedicated large polypectomy endoscopist assessed the post-EMR scar, while in 155 cases (13 %, 95 %CI 11 %–15 %), assessment of the post-EMR scar was performed by an endoscopist who was not specialized in large polypectomy (nondedicated). The cohort of hospitals was representative of the Dutch community hospital population.

### Post-EMR scar identification

A tattoo was placed in 488 /1277 cases (38 %, 95 %CI 36 %–41 %). In 1215/1277 cases (95 %, 95 %CI 94 %–96 %), the post-EMR scar was identified and assessed during surveillance colonoscopy. The presence of a submucosal tattoo was not associated with a higher identification rate of the post-EMR scar (95 % vs. 95 %, Bonferroni corrected *P* > 0.99) ( Table 2 ). Performance of scar inspection by a dedicated endoscopist instead of nondedicated endoscopist was independently associated with a higher scar identification rate (96 % vs. 88 %, Bonferroni corrected *P* < 0.001). The scar identification rate increased with increasing LNPCP size, from 92 % (95 %CI 89 %–95 %) in lesions of 20–29 mm, to 95 % (95 %CI 92 %–97 %) in lesions of 30–39 mm, and 98 % (95 %CI 96 %–99 %) in ≥ 40-mm lesions (overall Bonferroni corrected *P* = 0.03). Other lesion characteristics (proximal location, difficult accessibility, and flat morphology) did not significantly influence the scar identification rate.

**Table 2:** Factors associated with post-EMR scar identification 1 .

	Adjusted risk ratio	95 %CI	*P* value	Bonferroni corrected	
				95 %CI	*P* value
Dedicated endoscopist	1.82	1.35–2.47	**< 0.001**	1.20–2.77	**< 0.001**
Submucosal tattoo	1.04	0.82–1.32	0.72	0.75–1.45	0.99
**Size, mm**					
20–29	Ref		0.005		0.03
30–39	1.19	0.91–1.54	0.20	0.83–1.69	> 0.99
≥ 40	1.68	1.23–2.29	**0.001**	1.09–2.57	**0.008**
Flat morphology of initial LNPCP	0.88	0.68–1.13	0.31	0.62–1.25	> 0.99
Proximal location	0.84	0.65–1.09	0.19	0.59–1.20	> 0.99
Difficult accessibility	0.88	0.64–1.20	0.42	0.57–1.35	> 0.99

EMR, endoscopic mucosal resection; LNPCP: large nonpedunculated colorectal polyp.

1 Multivariable generalized estimating equation (GEE) analysis with correction for clustering of patients within endoscopist; intraclass correlation 0.014.

### Post-EMR scar assessment

All post-EMR scars were assessed using high definition white-light and advanced imaging, as validated by the presence of procedural images. In 1097 /1215 of the post-EMR scars (90 %, 95 %CI 88 %–92 %), the use of zoom or near focus could be confirmed by inspection of procedural images. Histology of the post-EMR scar was obtained from biopsies or treatment of recurrence in 1050 cases. The median number of biopsies was four (IQR 3–5).

The outcomes of optical assessment compared with histological confirmation by biopsy are presented in Table 3 . The overall prevalence of recurrence in this prospective cohort was 200/1050 (19 %, 95 %CI 17 %–22 %). The optical diagnosis of post-EMR recurrence showed a high diagnostic accuracy of 93 % (95 %CI 91 %–94 %), with a sensitivity of 93 % (95 %CI 88 %–96 %), specificity of 92 % (95 %CI 90 %–94 %), PPV of 74 % (95 %CI 68 %–80 %), and NPV of 98 % (95 %CI 97 %–99 %). The agreement between the optical assessment of recurrence and histological evaluation of biopsies was good, with a Cohen’s kappa of 0.78 (95 %CI 0.73–0.83). Intention-to-treat analysis, in which nonbiopsied post-EMR scars were included, with the assumption made that optical assessment and histology would both be negative for recurrence, showed similar results ( **Table 1 s** ).

**Table 3:** Outcomes of optical assessment and biopsy of post-EMR scars.

		Histology		
		Recurrence	No recurrence	
Optical assessment	Recurrence	186	64	250
	No recurrence	14	786	800
		200	850	1050
		**Outcome**	**95 %CI**	
Prevalence		19 %	17 %–22 %	
Sensitivity		93 %	88 %–96 %	
Specificity		92 %	90 %–94 %	
Positive predictive value		74 %	68 %–80 %	
Negative predictive value		98 %	97 %–99 %	
Diagnostic accuracy		93 %	91 %–94 %	
Cohen’s kappa		0.78	0.73–0.83	

EMR, endoscopic mucosal resection.

Sensitivity analysis, in which clustering of patients within endoscopists is taken into account, also showed similar results: sensitivity 93 % (95 %CI 90 %–96 %), specificity 93 % (95 %CI 90 %–95 %), PPV 74 % (95 %CI 68 %–81 %), NPV 98 % (95 %CI 97 %–99 %), and diagnostic accuracy 93 % (95 %CI 91 %–95 %).

The optical recurrence miss rate was 1 % (11/960; 95 %CI 0.6 %–2.0 %) for dedicated endoscopists and 3 % (3/90; 95 %CI 1.1 %–9.3 %) for nondedicated endoscopists ( *P* = 0.11).

### Influence of clip placement on post-EMR scar assessment

In 223/1050 histologically evaluated scars (21 %), clips were used to close the initial EMR defect. The PPV for optical diagnosis of recurrence in post-EMR scars decreased after clipping, from 78 % (95 %CI 72 %–84 %) in the nonclipped group to 63 % (95 %CI 50 %–74 %) in the clipped group. Risk regression accounting for clustering of patients within endoscopist (GEE intraclass correlation 0.004) on accuracy of optical recurrence assessment with correction for dedicated versus nondedicated endoscopists showed an RR for clipped versus nonclipped of 0.73 (95 %CI 0.58–0.90; *P* = 0.004). Furthermore, the proportion of false positives out of total assessments was higher after clipping (11 % vs. 5 %; *P* = 0.02).

### Certainty of post-EMR scar assessment

There was high certainty about post-EMR scar assessment performed by both dedicated and nondedicated endoscopists (95 % vs. 94 %, respectively; *P* = 0.71). In the post-EMR scars where endoscopists identified recurrence with certainty (n = 187), the PPV was 86 % (95 %CI 80 %–90 %), while this was only 41 % (95 %CI 29 %–54 %) in the scars where endoscopists were uncertain about the presence of recurrence (n = 63).

False-positive cases in the uncertain group (n = 37) were more often biopsied and less often endoscopically treated, compared with false-positive cases in the certain group (n = 27). Biopsies were performed in 20/37 cases (54 %, 95 %CI 37 %–70 %) in the uncertain group, compared with 9/27 cases (33 %, 95 %CI 17 %–54 %) in the certain group. Endoscopic treatment was performed in 17/37 cases (46 %, 95 %CI 30 %–63 %) in the uncertain group, compared with 18/27 cases (67 %, 95 %CI 46 %–83 %) in the certain group ( *P* = 0.09). Endoscopic treatment of false-positive cases was performed with re-EMR, cold snare polypectomy, cold avulsion with snare-tip soft coagulation (CAST), argon plasma coagulation, or hot snaring.

## Discussion

In this post-hoc analysis of the STAR-LNPCP study 9 , optical assessment of post-EMR scars for recurrence at 6 months was excellent, with a sensitivity of 93 % (95 %CI 88 %–96 %), specificity of 92 % (95 %CI 90 %–94 %), NPV of 98 % (95 %CI 97 %–99 %), and good agreement between optical assessment and histological confirmation, represented by a Cohen’s kappa of 0.78 (95 %CI 0.73–0.83). Dedicated endoscopists were more likely to identify the post-EMR scar (96 % vs. 88 %). Tattoo placement was not significantly associated with scar identification. Clipping of the post-EMR defect significantly complicated the correct optical assessment of the scar, as demonstrated by a higher number of false positives after clipping (11 % vs. 5 %).

In this study, scar identification was associated with the experience of the endoscopist, but not significantly with the placement of a tattoo, which argues against universal placement of a tattoo after EMR. A recent Delphi agreement report stated that a tattoo should be placed for polyps > 20 mm resected piecemeal with additional predictors of recurrence 11 ; there was an 84 % level of consensus. The following additional predictors were suggested: size > 40 mm, the use of adjunctive thermal techniques, size, morphology, site, access (SMSA) score of 4, and a prior failed attempt. This advice is grounded on the assumption that it is difficult to identify the scar in a significant number of cases, although clear data on the magnitude are limited 12 . A submucosal marking would support correct identification of the scar. However, a more recent study showed that application of easy-to-use criteria, such as a pale area, convergence of folds, and disruption of the normal colonic surface microvasculature, showed a scar identification rate of 99.7 % 5 7 . These results were obtained in high volume, experienced centers, which shows that identification is related to experience. Our study confirms these findings in a real-life practice setting. Scars of larger LNPCPs in particular were more easily identified because of more clearly visible features.

Taken together, these results provide an argument for a practice wherein endoscopists performing EMR evaluate the post-EMR scar themselves, instead of universally placing a tattoo. Tattoo placement has been shown to interfere with successful resection if the tattoo is aligned with the scar or a residual adenoma, can accidently be injected in the peritoneal cavity or mesorectum, and adds unnecessary costs. It should therefore be restricted to post-EMR scars that are anticipated to be difficult to identify.

On a national level, our outcomes are similar to those reported in tertiary centers. This is demonstrated by an NPV of 98 % and a PPV of 74 % in this national cohort, which is in line with the NPVs and PPVs in tertiary centers, as reported by the Australian ACE cohort (NPV 99 %, PPV 76 %) and ESCAPE trial (NPV 97 %, PPV 81 %) 5 6 . Clipping significantly complicated the optical assessment of post-EMR scars. With the increasing use of clips to prevent post-polypectomy bleeding, post-EMR scar clip artifacts will be increasingly detected 13 14 . The presence of a scar clip artifact decreased the PPV and increased the rate of false-positive cases, in which unnecessary endoscopic treatment was performed. Careful inspection of the post-EMR scar with advanced imaging should however lead to a correct differentiation between clip artifact and neoplastic recurrence.

Furthermore, the current study showed that uncertainty about the presence of recurrence led to biopsies being more frequently taken instead of direct treatment. The latter (i. e. optical assessment and treatment of a suspected recurrence in the same session) is advised in the current guidelines 2 15 . Although recurrence was less often observed in uncertain cases, overtreatment of non-neoplastic tissue outweighs postponing treatment of the recurrence to obtain histological confirmation, as it results in unnecessary additional costs and burden for the patient. The most used treatment modalities for recurrence (CAST, hot avulsion, cold/hot snare polypectomy) are known to have few complications. Furthermore, most recurrences have been shown to be small, unifocal, and easy to treat. While endoscopic overtreatment is not desirable, it should be noted that the risk of missing neoplastic recurrence is much more concerning than the overtreatment of non-neoplastic tissue. Therefore, treatment of any inconclusive nodules or areas in a post-EMR scar should still be performed. Additionally, when there is absolute certainty about the absence of recurrence, biopsies can be omitted.

This study is important because it adds to the growing evidence that optical diagnosis is highly accurate for the exclusion of post-EMR recurrence 16 17 . Data are obtained from a structured multicenter trial, on community level, with a large number of post-EMR scars. Therefore, results are considered generalizable to everyday colonoscopy practice. Obtaining an NPV of 98 % at a community level, clearly surpassing the PIVI (preservation and incorporation of valuable endoscopic innovations) threshold of 90 %, shows that, with high certainty optical diagnosis, standard biopsies can be omitted, resulting in significant cost reductions.

Several limitations should also be emphasized. The first limitation concerns the observed protocol violations in 13.5 % of cases in this study. According to the study protocol, all post-EMR scars should have been biopsied in a standardized manner; however, in 165/1215, this was not performed. This could have led to an overestimation of the diagnostic accuracy and NPV of optical assessment of the post-EMR scar, because of possible false-negative cases not being histologically confirmed in this cohort. Given the large number of cases in this cohort and the high NPV obtained with small CIs, it is however unlikely that these protocol violations would have significantly changed the outcomes.

A second limitation is that the follow-up is limited to the first surveillance colonoscopy at 6 months. As a result, it is possible that late recurrences may have been missed. It is known that approximately 4 % of patients still develop a recurrence despite showing a scar without recurrence at 6 months 3 18 . Routine biopsies are however unlikely to have a significant impact on this number of late recurrences. The biopsy protocol was extensive, with biopsies at the center and at the periphery, with a median number of four biopsies. Sampling error could have occurred but would be inherent to the implementation of routine scar biopsies, and therefore does not dismiss another follow-up endoscopy at 18 or 36 months after EMR.

A third limitation might be the effect of participating in a randomized controlled study on the performance of the endoscopist after training. An e-learning package was offered at the beginning of the study. Although the e-learning on scar identification and recurrence detection was offered to all participants to increase the quality of the study, and by itself was not part of the study intervention, it may have caused a learning effect. This may limit the generalizability of our results to an untrained group of community endoscopists. The uptake of the e-learning was 49 %. The NPV in the group of trained dedicated endoscopists was similar compared with the group of untrained dedicated endoscopists (99 % vs. 98 %). The effect of training is therefore likely to be limited. Furthermore, participating in a study may have caused a Hawthorne effect, increasing the performance of the participants, which would also limit the generalizability to real-life practice.

A fourth limitation is a lack of power owing to low numbers in the difference in optical recurrence miss rate between dedicated and nondedicated endoscopists. Furthermore, the frequent use of zoom/near focus may limit the generalizability of our study, as this may not be available in every hospital. Previous studies have shown that the use of zoom/near focus increases the detection rate of recurrence 5 6 . Lastly, post-EMR scar identification was performed by a dedicated endoscopist in the majority of cases in our study. This might also limit the generalizability of our results to the population of endoscopists working in community practice settings; however, this further underlines the importance of dedicated endoscopists performing post-EMR scar assessment.

In conclusion, the quality of optical assessment for recurrence at the post-EMR scar at a community level was found to be high. Identification of the post-EMR scar was high and the optical recurrence miss rate was low, especially from dedicated endoscopists. Therefore, routinely taking biopsies of the post-EMR scar could be omitted, as well as universal tattoo placement after EMR.

## Supplementary material

Supplementary material Zusatzmaterial
