# Supplementary material for: Optical assessment of scars after endoscopic mucosal resection of large colorectal polyps in a multicenter, community hospital setting: is routine biopsy still necessary?
Source: Endoscopy. 2025 Jan 28;57(6):620–8. doi: 10.1055/a-2498-7114 (PMC12119144; doi:10.1055/a-2498-7114)
Supplement: Supplementary file 1 — Supplementary material [file 23826supmat_10-1055-a-2498-7114.pdf]

Optical assessment of scars after endoscopic mucosal resection of large colorectal polyps in a multicenter, community hospital setting: is routine biopsy still necessary?

Lonne W.T. Meulen, Roel M.M Bogie, Peter D. Siersema, Bjorn Winkens, Marije S. Vlug, Frank H.J. Wolfhagen, Martine A.M.C. Baven-Pronk, Michael P.J.A. van der Voorn, Matthijs P. Schwartz, Lauran Vogelaar, Alaa Alkhalaf, Tom C.J. Seerden, Wouter L. Hazen, Ruud W.M. Schrauwen, Lorenza Alvarez Herrero, Ramon-Michel Schreuder, Annick B. van Nunen, Esther Stoop, Gijs J. de Bruin, Philip Bos, Willem A. Marsman, Edith Kuiper, Marc de Bièvre, Yasser A. Alderlieste, Robert Roomer, John Groen, Marloes Bigirwamungu-Bargeman, Monique E. van Leerdam, Linda B.J. Roberts-Bos, Femke Boersma, Karsten Thürnau, Roland de Vries, Jos M. Ramaker, Rogier J.J. de Ridder, Maria Pellisé, Michael J. Bourke, Ad A.M. Masclee, Leon M.G. Moons (on behalf of the OPTICAL-STAR study team)

CONTENTS

Appendix 1s: Description of topics discussed in biopsy e-module 1

Figure 1s: Examples from biopsy e-module 2

Table 1s: Outcomes optical assessment and biopsy of post-EMR scars intention-to-treat* 5

Appendix 1s: BIOPSY E-MODULE

Duration e-module: 26 minutes
Topics:

- Prevalence of post-EMR recurrence
- Steps in optical assessment of the scar → size, margins, presence of nodules, number of sites of recurrence, location of recurrence
- Value of advanced imaging and near focus/zoom
- Examples of local recurrence and ESCAs
- Value of biopsies of the scar + standard biopsy protocol.

Examples of the biopsy e-module are shown in **figure 1s**.

Endoscopy | DOI 10.1055/a-2498-7114 | © The Author(s).

Figure 1s Examples of the biopsy e-module.

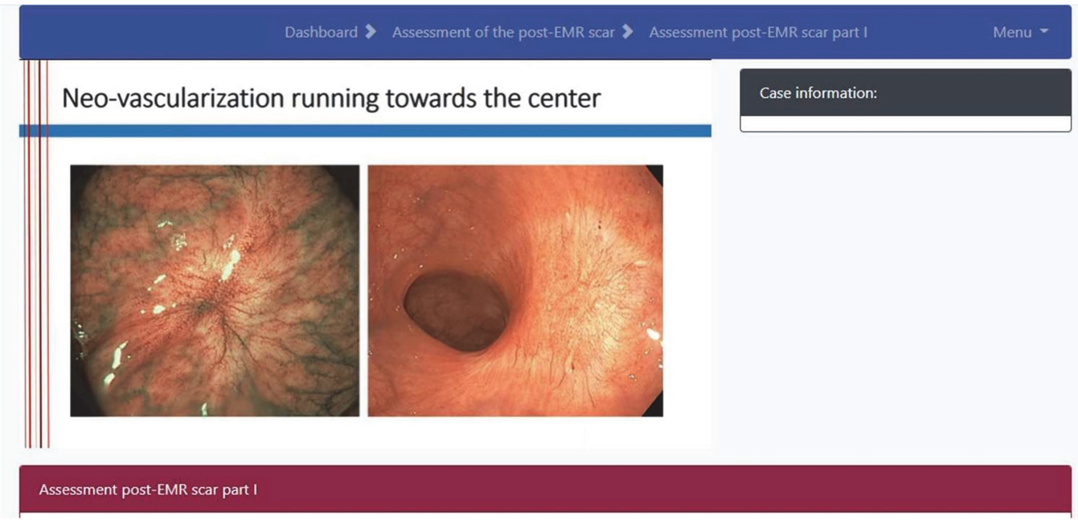

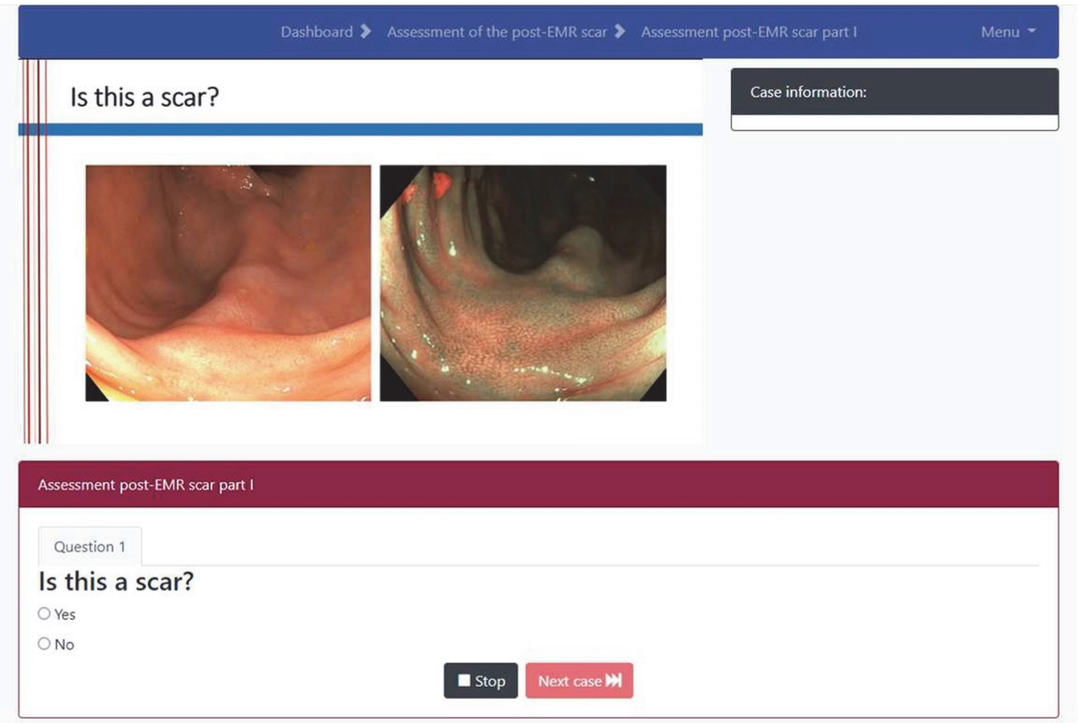

Endoscopy | DOI 10.1055/a-2498-7114 | © The Author(s).

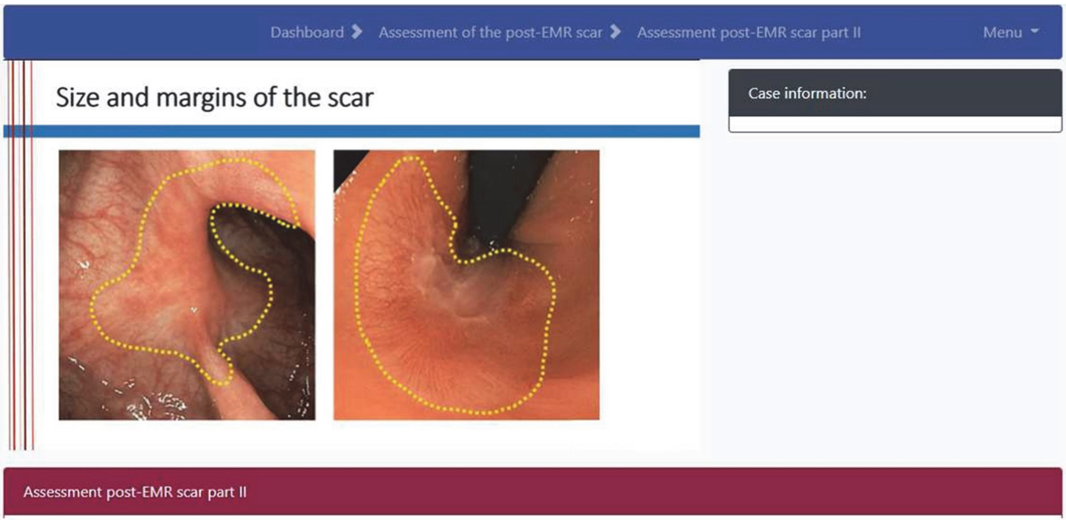
Dashboard
Assessment of the post-EMR scar
Assessment post-EMR scar part II
Menu
Size and margins of the scar
Case information:
Assessment post-EMR scar part II

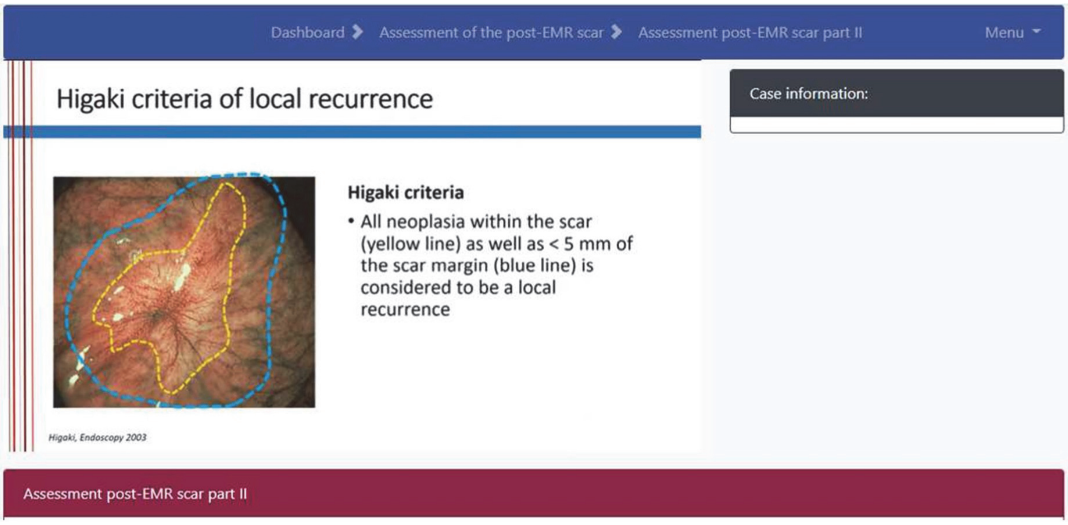
Dashboard
Assessment of the post-EMR scar
Assessment post-EMR scar part II
Menu
Higaki criteria of local recurrence
Case information:
Higaki criteria
• All neoplasia within the scar (yellow line) as well as < 5 mm of the scar margin (blue line) is considered to be a local recurrence
Higaki, Endoscopy 2003
Assessment post-EMR scar part II

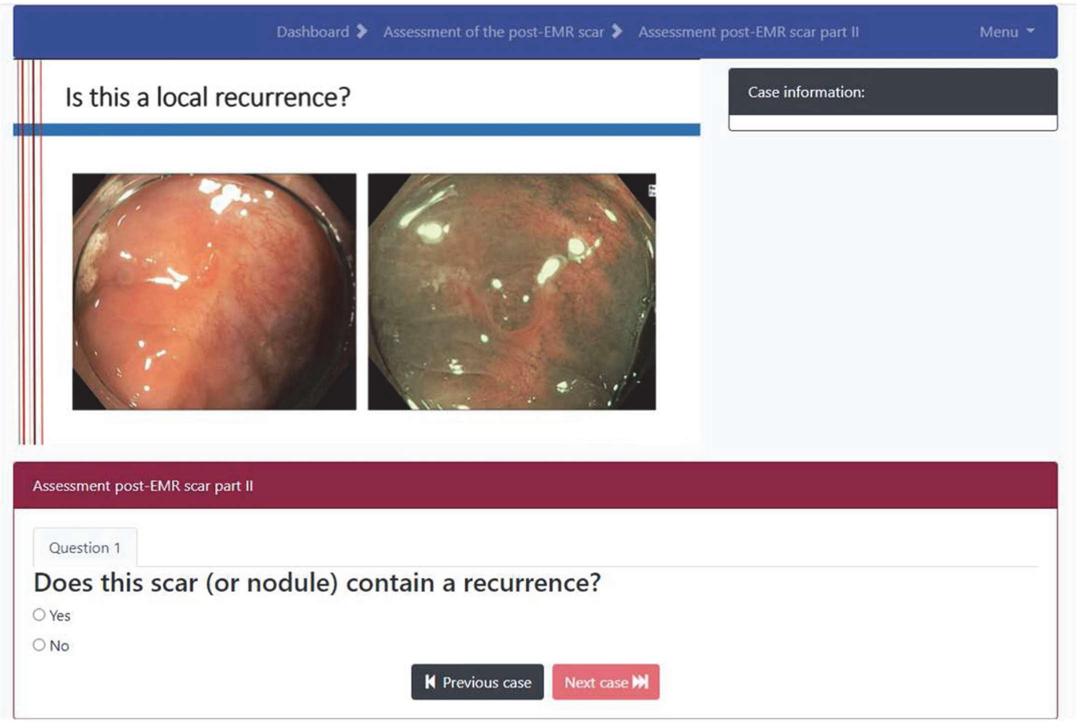

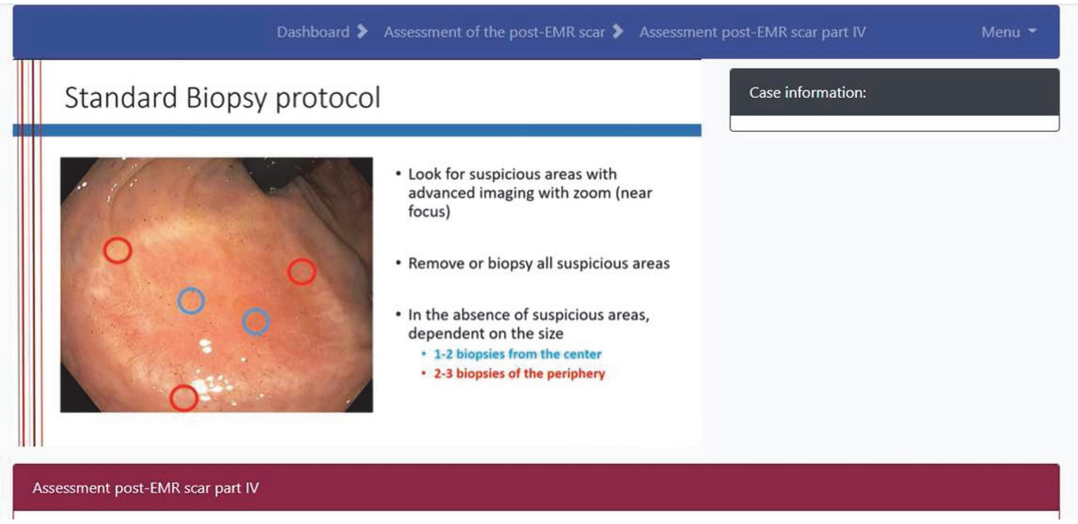

Endoscopy | DOI 10.1055/a-2498-7114 | © The Author(s).

Table 1s. Outcomes optical assessment and biopsy of post-EMR scars intention-to-treat*

		Biopsy		
		**Recurrence**	**No recurrence**	
**Optical assessment**	**Recurrence**	186	64	250
	**No recurrence**	14	951	965
		200	1015	1215

Prevalence	16% (14-19%)
Sensitivity	93% (88-96%)
Specificity	94% (92-95%)
Positive predictive value	74% (68-80%)
Negative predictive value	99% (98-99%)
Diagnostic accuracy	94% (92-95%)
Cohen's kappa (κ)	0.79 (0.74-0.83)

**Assuming that scars that were not found during colonoscopy did not reflect any signs of recurrence and non-biopsied scars would be confirmed histologically negative for recurrence if biopsy had taken place.*

Endoscopy | DOI 10.1055/a-2498-7114 | © The Author(s).
